# Validation of Simultaneous Biochip-based Method for Screening of 3 Beta-Lactam Families Residues in Cow’s Milk in Accordance with the European Union Decision 2002/657/EC and its Application on Real Samples

**DOI:** 10.22037/ijpr.2021.115441.15375

**Published:** 2021

**Authors:** Hassan Yazdanpanah, Mahraz Osouli, Elham Rashidi, Zakieh Karimi, Alireza Yazdanpanah, Sama Maani, Jamshid Salamzadeh, Arash Mahboubi, Samira Eslamizad

**Affiliations:** a *Food Safety Research Center, Shahid Beheshti University of Medical Sciences, Tehran, Iran. *; b *Department of Toxicology and Pharmacology, School of Pharmacy, Shahid Beheshti University of Medical Sciences, Tehran, Iran. *; c *Department of Medicinal Chemistry, Faculty of Pharmacy, Tehran Medical Sciences, Islamic Azad University, Tehran, Iran. *; d *School of Pharmacy, Shahid Beheshti University of Medical Sciences, Tehran, Iran.*; 1 *H. Y. and M. O. contributed equally to this work.*

**Keywords:** Beta-lactams, Milk, Multi-array, Biochip, Validation, Decision 2002/657/EC, Screening

## Abstract

Illegal and excessive use of veterinary antibiotics as a food additive for growth promotion in livestock can lead to allergic reactions and antibiotic resistance, which is a worldwide concern. A biochip-based semi-quantitative screening method of antimicrobial residues in milk was validated based on Commission Decision 2002/657/EC and the European guideline to validate screening methods for veterinary medicines. This multi-analytical screening method enables to determine of 3 beta-lactams (cefalexin, ampicillin, and cefuroxime) simultaneously. Analysis of 20 blank and 20 spiked milk samples showed that for all 3 antibiotic residues, the positivity threshold T was above cut-off value Fm, and no false-positive results were obtained for all 3 antibiotics. All detection capabilities (CCβ) were below Maximum Residue Level (MRL) authorized by European Commission. 47 UHT cow’s milk samples collected from Tehran province, IR Iran, were screened, and compliance was found in 100% of samples. This study found that the biochip method is valid to determine antibiotic residues in milk samples at the measured validation levels. The method was fast, simple, and able to simultaneous screen three families of beta-lactams from a single milk sample with almost no sample preparation.

## Introduction

Antimicrobials are widely consumed by animals for different purposes, including therapeutic, prophylactic, and sub-therapeutic effects for growth promotion and feed efficiency. More than 60,000 tons of antibiotics are used in animal husbandry per year (1, 2).

Most of the parent products of antibiotics and their metabolites are eliminated mostly via the urine and to a lesser extent in feces. However, after excretion, the portion of the drugs may remain as residues in milk, meat, and eggs (1). Antibiotic residues in raw milk can upset the dairy production process, and contamination of pasteurized milk causes allergic reactions and gastrointestinal problems in consumers (3). Two of the most important detrimental effects of overuse of antibiotics are hypersensitivity reaction and antibiotic resistance, which have significant public health consequences (4, 5).

US Food and Drug Administration, the European Union (EU), and other international regulatory authorities introduced some limitations for an acceptable amount of residues in foodstuffs of animal origin as maximum residue limits (MRLs) for some particular veterinary antibiotics used in animal husbandries to control and minimize the possible adverse effects of antibiotic residues on consumer health (6–9). MRLs were authorized by the EU for each compound in an individual matrix. The MRLs for beta-lactam antibiotics in milk are as follows: 100 µg/kg for cefalexin, 4 µg/kg for ampicillin, and there is no specific established MRL for cefuroxime. Therefore, the detection of the small amount of antibiotic residues in milk samples due to these MRLs requires very sensitive and selective analytical methods (5, 9).

The literature shows significant contamination of raw and pasteurized milk in Iran in different provinces due to inappropriate use of antibiotics in cattle and lack of compliance for spending withdrawal period by cattle farmers. (3)

There are different methods for detecting antibiotic residue, classified as chromatographic, microbiological, immunological, and miscellaneous. The most sensitive and specific methods are based on chromatographic techniques; nevertheless, immunological and microbiological methods are useful screening tools due to their lower cost, simplicity, and fast rate (1). This method is a multi-family method (multiplex) for 3 beta-lactams residues. In addition, detection limits of microbiological tests are often satisfactory for 3 beta-lactams residues in milk. Another advantage of the Βeta-Lactams antibiotics Array Plus kit is that ithere is no requirement for confirmatory method for negative results, so the confirmatory costs by LC-MS/MS are reduced. Up to 80 samples could be analyzed during one day with this kit.

This study reports the validation of an innovative multi-analytical system, Evidence Investigator based on biochip array technology, for the simultaneous determination of 3 beta-lactams -cefalexin (CEX), ampicillin (AMP), and Cefuroxime (CXM)- residues from a single cow’s milk sample. This methodology was validated based on European Decision No. CE/2002/657 and according to the European guideline for the validation of screening methods for the residues of veterinary medicines (12, 13). After validation, 47 UHT milk samples collected from Tehran were screened with this biochip-based technology to monitor these antibiotic residues.

## Experimental

*Chemicals and reagents*

CEX, AMP, and CXM were purchased from Sigma-Aldrich (Germany). Βeta-Lactams antibiotics Array Plus kit (EV 3957A/B) and Milk Preparation Kit (EV 3776) were from Randox Food Diagnostics (UK).

*Apparatus*

Vortex model Hei-MIX Reax top (Heidolph, Germany), centrifuge Rotinta 380R (Hettich, Germany), roller mixer model BMW-4-1-10-R-1-89 (Behdad, IRAN), and Evidence Investigator biochip analyser (Randox Food Diagnostics, UK) were used in this study.

*Blank and real milk samples*

Twenty different batches of blank milk were selected with varying degrees of fat and shelf life. Samples of milk were collected from Austria and the UK and analyzed Using Biochip Array Technology to ensure they did not contain any residues of the 3 antibiotics. Forty-seven UHT treated and homogenized milk samples were bought from retail outlets from July to August 2017. The shelf life of UHT milk samples at room temperature is 6 months. These samples were collected from Tehran city and stored at 2-8 ºC for 4-5 months.

*Preparation of standard solutions*

Standard stock solutions of all antibiotics were made at a concentration of 1 mg/mL in methanol. To prepare the intermediate standards, the stock solutions were diluted with methanol resulting in concentration of 10 ng/mL and in the same way working solutions were made. 

*Sample preparation*

One hundred microliters of the working standard solution were diluted by 900 µL of blank milk to make spiked samples. The blank milk samples were spiked with the mix solution at three levels for each compound (1 µg/kg for CEX, 2, µg/kg for AMP, and 3 µg/kg for CXM).

Before analysis on the biochip platform, there is no particular sample preparation for milk samples except one-step centrifugation (10 min at 2880 rcf) for semi-skimmed and full-fat milk samples. 

*Evidence Investigator system*

*Multi-array biochip technology*

Beta-Lactams Array Plus kit applied to the Evidence Investigator biochip analyzer was used (Randox Food Diagnostics, UK.).

The base of the Evidence Investigator system is a biochip that contains an array of discrete test regions (DTRs) of collected antibodies at each spatially distinct DTR. For simultaneous detection of beta-lactams, a competitive chemiluminescent immunoassay format is employed. Horseradish peroxidase (HRP) labeled conjugate is used. Increased levels of antimicrobial in a sample lead to a decreased rate of binding of antimicrobial labeled with HRP and a decrease in the emitted signal (in Relative Light Unit, RLU), consequently (14, 15).

Each biochip carrier contained nine vessels where the immunoreactions are accomplished for individual samples. The analyses were performed according to the manufacturer’s instructions. Concisely, 100 µL of assay diluent and 100 µL of calibrator/sample were pipetted respectively in each biochip well. All edges of the handling tray (with the capacity to accommodate 6 carriers) were gently taped for mixing reagents. Then the handling tray was incubated at +25 °C and 370 rpm for 30 min in the thermoshaker provided. One hundred microliters of working strength conjugate were then added to each biochip followed by an incubation of 60 min at +25 °C and 370 rpm. Afterward, quick wash cycles were carried out, and after the final wash, any residual wash buffer was removed. The next working signal reagent (250 µL) was added to each biochip, which was shielded to protect from light. After precisely 2 min (± 10 s) the biochip carrier was located into the Evidence Investigator system and images were captured by the software.

*Image and data processing*

The base of biochip detection is a chemiluminescent signal by a CCD (charge-coupled device) camera, which records the light emission from the entire distinct test sites on each biochip simultaneously. The system includes dedicated software for processing and archiving the multiple data generated. The analyzer uses image processing software to quantify the RLUs) and analyte concentration (ppb). 

*Validation procedure*

According to European guideline and European Decision No 2002/657/EC, investigation of practicability, applicability, specificity, CCβ, and stability is required for validation of screening methods for residues of veterinary medicines (12).

*Number of samples required for validation*

As stated by the European guideline for determining the screening target concentration at half the Regulatory/Action Limit or lower (*e.g.* ½ MRL), at least 20 “screen positive” are needed to prove that CCβ is less than or equal to the ½ MRL.

*Identification of the Cut-Off Level and calculation of CCβ*

For recognizing a sample as a ‘screen positive’ or not in the validation of a qualitative or semi-quantitative screening method, determining a cut-off value is necessary. The cut-off level and CCβ were defined for the 3 beta-lactams. MRL, calibration range, and spiking level are represented in Table 1 (11, 13).

The average value (in RLU) and the SD of the signal of the 20 blank and 20 spiked samples at mentioned concentrations were calculated for each antibiotic. 

The threshold value T is calculated according to Equation 1:

T = mean RLU signal of the blank – 1.64×SD RLU signal of the blank Equation 1.

The cut-off factor Fm was calculated from the samples spiked with 3 antibiotic residues (CEX, AMP, and CXM) as follows:

Fm = mean RLU signal of the spiked samples + 1.64 × SD RLU signal of the spiked samples 

Depending on the T value in comparison to the Fm (if Fm is beneath the T value), the target concentration during the validation is selected as CCβ; otherwise (if the T value is beneath Fm), the concentration of antibiotics in the validation step should be increased.

*Practicability*

The purpose of the study on practicability was to investigate whether the method is capable or not for routine analysis. The simplicity of analyzing, the need for usual laboratory equipment, instruments, and conditions in the validation procedure all show the practicability of this screening method.

*Applicability*

The applicability of the kit and method for screening 3 different antibiotic families was checked with different types of milk samples (low fat, semi-fat, and full-fat) and storage duration from different sources. 

*Stability*

The stability of antibiotic residues in milk was noticed based on a literature review.

*Application of this method on real samples*

Real samples of UHT treated and homogenized cow’s milk samples (n = 47) were examined to determine the presence of 3 antibiotic families simultaneously.

## Results

*Detection capabilities*

**Figure 1:**
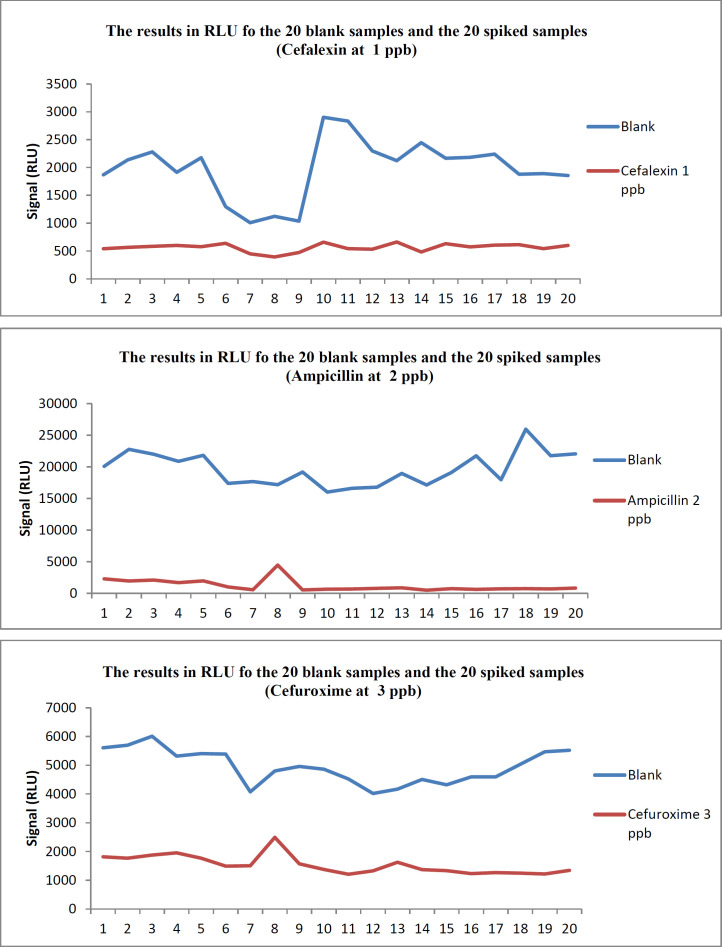
The results in RLU for the 20 blank and the 20 spiked samples for Cefalexin, Ampicillin and Cefuroxime

The distribution of the screening results for 20 blank and 20 spiked samples with the three antibiotic residues CEX, AMP, and CXM is presented in Figure 1. On the first day of the validation procedure, all the samples spiked at the levels shown in Table 1 were detected. The results obtained when Fm was selected as the cut-off value are summarized in Table 2. 

An acceptable rate of false-negative results of 5% was obtained for 3 beta-lactams, 1 out of 20 for AMP, and 1 out of 20 for CXM. Therefore due to the percentages of false-negative results, the validated concentration was considered as CCβ**.** According to Commission Decision 2002/657/EC (13, 14), the screening target concentration for mentioned analytes is at or below the regulatory limit (MRL), the chosen spike levels (Validation concentration) were selected as CCβ as shown in Table 3.

*Practicability*

No specific sample preparation was needed. Only semi-skimmed and full-fat milk samples need one step centrifugation before applying to the biochip. One hundred microliters of each sample was required for testing.

The kit contained enough amount of material, and the procedure was simple to do. The software were very user-friendly. The results were shown in ppb and RLU. The data disk was present in each box separately. 

*Specificity and false-positive rate*

Twenty blank and 20 spiked milk samples were analyzed during 3 days in the validation procedure. When T was set as the cut-off value, 2 samples out of 20 (10%) were screened as false-positive for CEX, and no false–negative screening results were observed (Table 4); the method would be more sensitive, but increasing of the false-positive ratio would cause to costly confirmatory analyses. Taking Fm as the cut-off level is a modification between detection capabilities, low enough to reach the respective recommended concentration (RC) and an acceptable false-positive rate. So, Fm was set as the cut-off value to decide on the positivity of a sample (Table 3 shows the CCβ; Table 2 shows results when Fm is taken as a cut-off). 

*Applicability*

Variation between fat content and storage duration of real milk samples from different sources did not affect the results, indicating that the Beta-Lactams Array Plus kit is appropriate to a wide range of the samples.

*Stability of antibiotic residues*

The stability of CEX, AMP, and CXM residues in milk was reported in some studies. In one study, the stability of five beta-lactam antibiotics, including ampicillin in bovine milk, was investigated, and the findings showed that ampicillin was stable over 14 days at -18°C (16). Another study noticed higher ampicillin stability among other antibiotics (~24-35 weeks at -18°C and ~ 6 days at 4°C) (17). In another study, it was found that cefalexin and cefuroxime, according to the degradation criterion of 10%, were stable for 21 and 14 days at 4°C respectively (18). 

*Analyses of real milk samples*

The data collected from the analysis of 47 real milk samples with the Beta-Lactams Array Plus are presented in Table 5. The results showed that all 47 samples were presumptive negative for all 3 compounds.

## Discussion

Inappropriate usage of antibiotics in animals, such as prevention and treatment of infection and as a food additive for growth promotion, leads to harmful effects on human health (1, 19). 

Antibiotic residues could cause antibiotic hypersensitivity and antibiotic resistance in humans. Nowadays, a global health threat is the antimicrobial resistance, and for this reason, countries focused on restricting the use of antibiotics in animals and monitoring antibiotic residues in animal products such as milk (19, 20). 

Various screening and confirmatory methods for detecting and determining antibiotic residues in milk are presented. High technology and expensive chromatography methods, such as high pressure liquid chromatography (HPLC) and mass spectrometry (MS) are the common confirmatory methods. Because of the low cost and rapid of screening methods, they are used as the first-line method. Most of the screening tests are based on microbiological and immune assays methods (21). Examples of the available screening methods for screening beta-lactams residues in milk are shown in Table 6 (22).

In this study, the Beta-Lactams Array Plus kit was validated based on the Commission Decision 2002/657/EC and the European guideline to validate screening methods for veterinary medicines (12, 13). The results showed that this kit could be used as a valid screening method for the simultaneous determination of three families of beta-lactam antibiotic residues from a single milk sample at the validated levels. CCβ values were beneath the MRLs authorized by the European Commission. The method was found to be fast, simple, and safe. Different types of milk can be screened and no sample preparation procedure (or just one-step centrifugation) is required. 100% of the real samples screened were compliant for these 3 types of antibiotic residues. 

Many studies related to antibiotic residue detection in milk samples have been performed worldwide using different methodologies. In an analysis of ß-lactam antibiotic residues in milk on the Croatian market, among 105 milk samples, none showed the presence of ß-lactams (ampicillin, amoxicillin, benzylpenicillin, cephapirin, cloxacillin, cefoperazone, cefazolin, and ceftiofur) by TwinsensorBT Milk test® (23). The analysis of 973 milk samples, collected throughout the Netherlands, found 9 positive samples for beta-lactam antibiotic residues using a microbiological multiple system (24). In a survey, 192 samples of raw milk, collected in Niger, were tested for antimicrobial residue by Delvotest®, and 19 (9.9%) were positive (25). Studies in where 127 samples of raw milk, collected from Kosovo dairies, were screened by ELISA reported that 50.4% were contaminated with beta-lactams (26, 27). The assessment of local milk samples (collected in Kuwait) and imported milk samples for beta-lactam antibiotic residues showed that 62 out of 308 local raw milk samples, 8 out of 209 local pasteurized milk samples (full cream, low fat, and skimmed milk), and 28 out of 313 imported pasteurized milk samples, were above the MRL by using Charm II system (28). Penicillin G, amoxicillin, and cephapirin were present in 26, 3, and 2 milk samples, respectively out of 53 milk samples found presumptive positive with the microbial method Delvotest SP (29). In a survey in New York State, beta-lactams were detected in 75% of 34 waste milk samples collected from dairy farms after screening with a commercial enzyme-linked receptor-binding assay (30).

In Iran, a study reported the presence of beta-lactams in 4.66% of 150 UHT milk samples, collected from Tabriz milk stores, when tested with a BetaStar screening kit (31). In another study, the milk powder samples (240), collected from Tehran dairy factories were assessed with the BetaStar Combo; 30% of the whole samples were positive for beta-lactams (32). Forouzan *et al.* analyzed 848 pasteurized milk samples from North West of Iran (West Azerbaijan province) by Copan test kit for detecting beta-lactam antibiotics, tetracycline, and sulfonamides and 30.14% of samples were contaminated (33). Our study found 47 UHT treated and homogenized milk samples compliant when screened with Beta-Lactams Array Plus kit. 

The variety of detection methods for beta-lactams and the number of studies in this field indicate the importance of milk safety and quality worldwide suggesting the importance of monitoring antibiotic residues in different types of milk in Tehran and other provinces of IR Iran.

**Table 1:** Maximum Residue Limit (MRL), calibration range, and spiking level of the 3 antibiotics

**Compounds**	MRL (EU)* **(ppb)**	**Calibration range (ppb) regarding dilution factor**	**Chosen spike level** **(ppb)**
Cefalexin (CEX)	100	0-5	1
Ampicillin (AMP)	4	0-6	2
Cefuroxime (CXM)	-	0-20	3

*European Union.

**Table 2:** The summary results when Fm is considered as the cut-off value

	**CEX**	**AMP**	**CXM**
Concentration (µg/kg)	1	2	3
T value (RLU)	1116.28	15319.96	3980.66
Fm value (RLU)	679.92	2792.20	2076.48
T > Fm	Yes	Yes	Yes
Number of FP	0	0	0
FP rate (%)	0	0	0
Number of FN	0	1	1
FN rate (%)	0	5	5

RLU: Relative Light Unit; CEX: Cefalexin; AMP: Ampicillin; CXM: Cefuroxime, FP: false positive, FN: false negative.

**Table 3:** Detection capabilities CCβ

	**CEX**	**AMP**	**CXM**
LOD (ppb) (as per manufacturer)	0.3	0.75	1.25
Spike level used for validation (ppb)	1	2	3
CCβ (ppb)	1	2	3

CEX: Cefalexin; AMP: Ampicillin; CXM: Cefuroxime; LOD; Limit of Detection.

**Table 4:** Sum of false-negative and false-positive screening results when T was chosen as cut-off value

		**CEX**	**AMP**	**CXM**
T value (in RLU) (n = 20)		1116.28	15319.96	3980.66
Cut-off = T	false-positive	2	0	0
	false-negative	0	0	0
Fm value (in RLU) (n = 20)		679.92	2792.20	2076.48
Cut-off = Fm	false-positive	0	0	0
	false-negative	0	1	1

RLU: Relative light Unit; CEX: Cefalexin; AMP: Ampicillin; CXM: Cefuroxime, FP: false positive, FN: false negative.

**Table 5:** Occurrence of beta-lactams in UHT treated and homogenized milk samples in RLU

**Parameters**	**CEX**	**AMP**	**CXM**
Number of samples	47	47	47
Cut-off	679.92	2792.20	2076.48
Number of positive samples	0	0	0
Positive samples (%)	0	0	0

CEX: Cefalexin; AMP: Ampicillin; CXM: Cefuroxime.

**Table 6:** Comparison of various commercial kits or the screening methods of antibiotic residues in milk

**Commercial kit (manufacturer, country)**	**Principle of the test (Type of reaction)**	**Number of** **tested** **antibiotic** **residues** **(beta-lactams)**	**LOD of Cefalexin (μg/kg)**	**LOD of Ampicillin (μg/kg)**	**LOD of Cefuroxime** **(μg/kg)**	**Time per analysis**
BETASTAR®	Lateral flow	13(13)	-	2-5	-	5 min
BETASTAR® COMBO	Lateral flow	16(13)		2-3		5 min
BRT MRL-Screening Test / BRT Inhibitor Test	Microbial inhibition	30(8)	100 – 200 / 200 – 300	2-3 / 2-3	100 – 200 / 200 – 300	2 h to 2 h and 30 min
BR-Test AS Special	Microbial inhibition	14(5)	20 – 30	2-3	-	Starting from 2 h
BR-Test AS Brilliant	Microbial inhibition	14(5)	25-50	3-4	-	Reading time: 2 h and 45 min Control time: starting from 1 h 45 min
CHARM II BETA-LACTAM TEST	Radio-labelled Assay	12(12)	-	3-4	-	Approximately 12 minutes
CHARM BLUE-YELLOW TEST	Microbial inhibition	10(6)	-	5	-	Approximately 2 h and 45 min
CHARM TEST BSDA	Microbial inhibition	9(6)	-	6.7	-	Approximately 2 h and 45 min
CHARM COWSIDE TEST	Microbial Inhibition	12(6)	-	5	-	Approximately 2 h and 30 min
Copan Milk Test (CMT)	Microbial Inhibition	43(15)	50* 60-70**	2* 4**		Fixed time of 3 h
Delvo-X-press	Receptor assay	22(22)	25-50	4-8	4-20	7 min
Delvotest SP- NT	Microbial Inhibition	19(8)	-	4* 6-7**	-	Reading time: 3 h Control time: starting from 2 h and 15 min Delvotest® Accelerator: 100-105 min
Delvotest P/ Delvotest SP	Microbial Inhibition	26(13) / 31(13)	40-60* 60-100**	2-3* 3-5**	-	Analysis time: 2 h and 30 min/3 h Control time: starting from 2 h and 15 min
ECLIPSE FARM / ECLIPSE 50	Microbial Inhibition	16(7) / 28(10)	75	5	-	2.15-2.30 h
Euroclone KALIDOS TB	Microbial Inhibition	27(7)	-	4	-	3 h
Parallux	Solid phase immunoassay	14(6)	-	2.9	-	4 min
Penzym®100	Lateral flow	12(12)	20-40	4-7	50-100	15 min
PENZYM®100 S	Lateral flow	12(12)	15-25	3-4	30-60	22 min
ROSA MRL Test for Beta-Lactam	Lateral flow	14(14)	30-60	3-4	3-5	8 min
ROSA MRL3 Test for Beta-Lactam	Lateral flow	14(14)	10	4	-	3 min
SCREENING PLUS	Microbial Inhibition	16(4)	80	5	-	3.15-3.30 h
SNAP Test Kits - IDEXX	Lateral flow	20(15)	14-29	3.5-5	-	10 min
Twinsensor BT	Lateral flow	17(14)	-	3-5	-	6 min
Valio T 101 test	Microbial Inhibition	32(9)	50 -100	10 - 30	-	4 h and 30 min

^*^At control time (The control time is the time when the blank sample turns from purple to yellow).

^**^ about 2 to 3 h incubation (at 64 ± 1 °C in a water bath)

## Conclusion

To our knowledge, this is the first time that the Beta-Lactams Array Plus kit has been validated for milk samples based on the European guideline. Antibiotic residues in milk can cause harmful effects that lead to antimicrobial resistance and hypersensitivity but also have other implications such as major financial harms for producers, manufacturers of milk and milk products, and even governments. The validated method was created to be fast and screen 3 families of beta-lactams simultaneously in various kinds of milk, with the least sample preparation process. Although the real samples assessed in this survey were compliant, it is recommended that more samples of various types of milk from all provinces in IR Iran should be monitored to ensure safety and quality.
